# Starting a Fire Without Flame: The Induction of Cell Death and Inflammation in Electroporation-Based Tumor Ablation Strategies

**DOI:** 10.3389/fonc.2020.01235

**Published:** 2020-07-28

**Authors:** Rebecca M. Brock, Natalie Beitel-White, Rafael V. Davalos, Irving C. Allen

**Affiliations:** ^1^Graduate Program in Translational Biology, Medicine, and Health, Virginia Polytechnic Institute and State University, Roanoke, VA, United States; ^2^Department of Biomedical Engineering and Mechanics, Virginia Polytechnic Institute and State University, Blacksburg, VA, United States; ^3^Department of Electrical and Computer Engineering, Virginia Polytechnic Institute and State University, Blacksburg, VA, United States; ^4^Department of Biomedical Sciences and Pathobiology, Virginia-Maryland College of Veterinary Science, Blacksburg, VA, United States

**Keywords:** cancer, apoptosis, necrosis, pyroptosis, calcium, electroporation, ablation

## Abstract

New therapeutic strategies and paradigms are direly needed for the treatment of cancer. While the surgical removal of tumors is favored in most cancer treatment plans, resection options are often limited based on tumor localization. Over the last two decades, multiple tumor ablation strategies have emerged as promising stand-alone or combination therapeutic options for patients. These strategies are often employed to treat tumors in areas where surgical resection is not possible or where chemotherapeutics have proven ineffective. The type of cell death induced by the ablation modality is a critical aspect of therapeutic success that can impact the efficacy of the treatment and systemic anti-tumor immune system responses. Electroporation-based ablation technologies include electrochemotherapy, irreversible electroporation, and other modalities that rely on pulsed electric fields to create pores in cell membranes. These pores can either be reversible or irreversible depending on the electric field parameters and can induce cell death either alone or in combination with a therapeutic agent. However, there have been many controversial findings among these technologies as to the cell death type initiated, from apoptosis to pyroptosis. As cell death mechanisms can impact treatment side effects and efficacy, we review the main types of cell death induced by electroporation-based treatments and summarize the impact of these mechanisms on treatment response. We also discuss potential reasons behind the variability of findings such as the similarities between cell death pathways, differences between cell-types, and the variation in electric field strength across the treatment area.

## Introduction

Despite improvements in survival and quality of life provided by current therapeutic practices, cancer death rates remain unacceptably high (1). New treatment paradigms are direly needed. Minimally invasive tumor ablation treatments such as cryotherapy, laser irradiation, microwave irradiation, radiofrequency ablation, high-intensity focused ultrasound ablation, and irreversible electroporation (IRE), have shown significant promise (2, 3). Ablation modalities function through the direct or indirect induction of cell death, resulting in the destruction of tissue by thermal, mechanical, or electrical means. Thermal ablation modalities, such as microwave or radiofrequency, use intense temperatures to lyse cells and induce apoptosis in treatment margins (4). Unfortunately, tumor location near critical structures can lead to hemorrhaging, heat sink effects, and healthy tissue damages during treatment (5, 6). Non-thermal modalities such as those that utilize electroporation can overcome these treatment barriers to induce cell death through different mechanisms and may initiate systemic immune responses to target both the local tumor microenvironment and metastatic sites (7–10).

Extensive investigations have explored the mechanisms underlying cell death induced by pore formation and homeostasis loss following electroporation-based tumor ablation treatments (11, 12). However, it should be noted that thresholds for electroporation, both reversible and irreversible, and cell death induction can differ based on cell-type (13–16). This may be why studies utilizing electroporation-based treatments report different cell death mechanisms ranging from apoptosis to pyroptosis (8, 17). Cell death is a complex and nuanced process, especially in the context of cancer. The type of cell death induced can have significant biological and physiological consequences in terms of local and systemic treatment efficacy. Here, we review these four major types of cell death, offer insight into the biological impacts of each on tumor ablation, and summarize cell death findings from electroporation-based treatment strategies.

## Cell Death Mechanisms in Electroporation-Based Treatments

**Figure 1:**
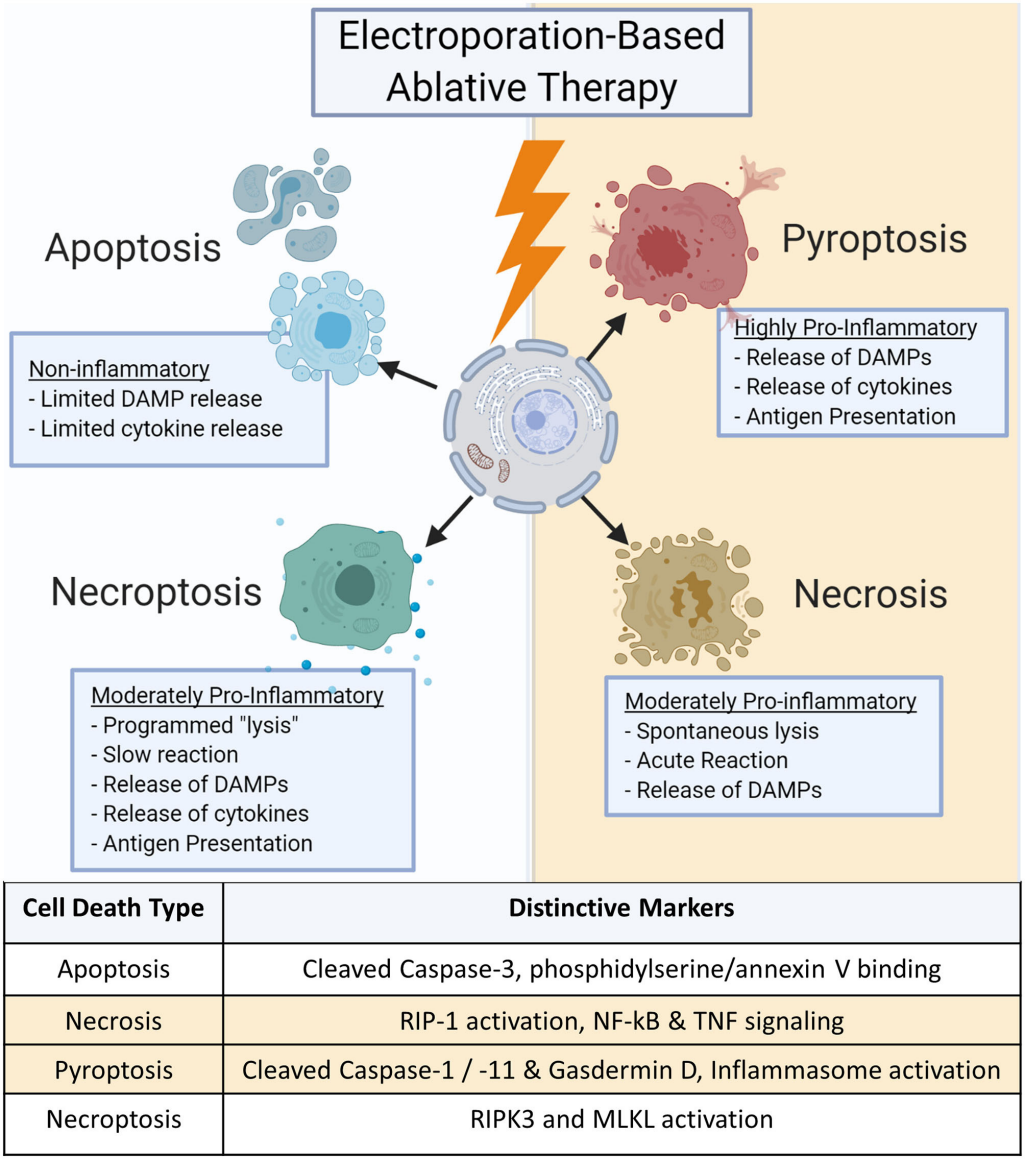
Cell death types initiated by electroporation-based ablation.

Cell death can diverge into many pathways, eliciting a large range of responses (Figure 1). Each pathway can be further modified by genetic, epigenetic, and regulatory factors for different cell types and tissues. These pathways are not singular; many similar proteins and biochemical pathways have been shown to be involved with multiple cell death subroutines, making it difficult to ascertain a definitive type of cell death (18, 19). Inducing the optimum type of cell death is critical for effective tumor treatment as it can influence both local and systemic effects that significantly impact cancer recurrence, inflammation, and autoimmunity (20–24).

### Apoptosis

Apoptosis is one of the most commonly mentioned forms of cell death in electroporation-based ablations (25–28). Generally considered non-inflammatory, apoptosis is a programmed form of cell death required for normal maintenance of tissues such as intestinal epithelium or epidermis where cells are regularly replaced to avoid the accumulation of cellular damage or mutations (29–31). This pathway is commonly dysregulated in cancers as the cells lose the ability to respond to internal signals due to mutations in apoptosis regulatory pathways or key genes such as Bcl2 or p53 (32, 33). The induction of apoptosis implies a “quiet cell death” with little immune involvement beyond dead tissue clearance. Hallmark features include the cleaving and activation of Caspase 3 and 7 along with the expression of phosphatidylserine on the cellular surface that binds Annexin V (34–36). In tumor ablation, apoptosis can be considered beneficial due to the reduced potential for inflammation-driven damage to nearby healthy tissues as it leads to the suppression of inflammatory signaling (37). While apoptosis can be highly effective in ablating primary tumors, the lack of innate and adaptive immune system activation is sub-optimal for inducing systemic anti-tumor immune responses. This can negatively impact the potential to induce an abscopal effect to target metastatic lesions and may create a permissive niche for tumor reoccurrence once the apoptotic pressure is removed (38, 39).

### Necrosis

Necrosis lies opposite of apoptosis and involves rapid cell death. Necrosis is typically induced by sudden loss in cell homeostasis, such as rapid osmolarity or temperature changes, influx of calcium into the cell or mitochondrial spaces, or mechanical tissue damages that can lead to autolysis (40). It should be noted that this section refers to cellular necrosis, which differs from “tissue necrosis,” or irreversible tissue injury, the presence of dead tissue often based on acellularity or tissue morphology and does not specify specific cell death mechanisms.

Necrosis is often referred to as accidental or lytic cell death and is characterized by the breakdown of the cell membrane and the release of large amounts of damage-associated molecular patterns (DAMPs) (38, 41). Further investigation into necrosis's mechanisms show that contrary to the chaotic description, its mechanism may also be regulated by specific programs similar to apoptosis (41). For example, the serine/threonine kinase RIP1 appears to play a pivotal role as a central initiator of necrosis (41). Activation of RIP1 results in NF-κB and transient MAPK signaling, directly inducing the production of pro-inflammatory mediators (42–44). RIP1 may also play a role in the TNF signaling and the generation of ceramide during necrosis (45). Likewise, calcium and reactive oxygen species (ROS) signaling cascades lead to the propagation and execution phases of cell death. Ultimately, these result in damaged proteins, lipids, and DNA that drive necrosis and are released upon cell death (41, 46). The production of DAMPs stimulate neighboring cells to activate the innate immune system, resulting in inflammation (47). Unfortunately, inflammatory signaling recruits immune cells to the lesion that may be polarized to facilitate regeneration growth and repair which the tumor can reprogram to assist the tumor in grow, repair, and create a more favorable niche for progression (48, 49). Necrosis may also induce tumor lysis syndrome (TLS) as large numbers of DAMPs acutely released into the blood stream can lead to systemic inflammation (50–52). Any tumor ablation modality that induces high DAMP production should focus on optimizing targeting and minimizing the treatment area. It should be noted that TLS has not been reported in electroporation-based treatment clinical trials.

### Pyroptosis

Pyroptosis is one of twelve identified subclasses of regulated cell death and displays high local inflammatory responses distinct from necrosis (36). Traditionally, pyroptosis is associated with the host innate immune response to viral and bacterial pathogens (53–56). Pyroptosis is an extremely specific form of inflammatory programmed cell death and is characterized by the cleavage and activation of Caspase-1 and Caspase-11 (55, 57). Caspase-1 activation results in the cleavage and processing of IL-1β and IL-18, potent proinflammatory cytokines. These caspases also cleave gasdermin D, generating an N-terminal cleavage product that drives pyroptosis (58). In addition to IL-1β and IL-18, pyroptosis produces a significant amount of DAMPs, including HMGB1, ATP, and ROS to further stimulate the innate immune system (56, 59, 60). This high signaling state leads to rapid responses from the body with recruitment of immune cells to the local area as well as increased systemic signaling to enhance immunosurveillance and heighten antigen presentation potential that could be beneficial for cancer treatment (61). While this can be ideal for allowing the immune system to recognize tumor cells in the body and create immune memory, the heightened inflammatory state can lead to severe side effects such as fever and autoimmunity (21, 51).

### Necroptosis

In addition to pyroptosis, a fourth major regulated cell death routine termed necroptosis has also been described following irreversible electroporation. Necroptosis is also termed programmed necrosis or alternative necrosis and has features characteristic to both apoptosis and necrosis. Necroptosis is triggered by perturbations of extracellular or intracellular homeostasis and does not induce a quick, automatic lysis of the cell (62). Rather, the cell produces low levels of DAMPs and proinflammatory cytokines that drive moderate levels of inflammation compared to much more inflammatory mechanisms associated with pyroptosis (19). Necroptosis critically depends on the pseudo-kinase mixed lineage kinase domain-like (MLKL) protein, which is phosphorylated by the kinase RIPK3 (63). While mechanistically less clear, RIPK1 has also been suggested to mediate necroptosis under some conditions and the activation of an inflammasome has been suggested to underlie inflammatory cytokine production (36, 64). This can make it somewhat challenging to discern from necrosis. However, due to the temporal nature of necroptosis and the attenuated production of local cytokines, necroptosis is less likely to induce TLS but still retains the potential of inducing inflammation that can promote an anti-tumor microenvironment and improve antigen presentation.

## Electroporation-Based Ablation Modalities

### Electrochemotherapy (ECT) and Calcium-Based Electroporation

ECT was one of the first electroporation-based modalities to progress to clinical trials and is routinely used in Europe for cutaneous malignancies at over 150 centers (65–67). ECT utilizes pulsed electric fields (PEFs) of micro- to nanosecond pulse durations for short periods that lead to reversible electroporation and allow for the internal delivery of chemotherapeutic reagents to the site of treatment (68, 69). Many chemotherapeutic agents such as bleomycin or cisplatin can have off-target effects, difficulty penetrating beyond the surface of the tumor, and the potential for chemoresistance (70, 71). Thus, ECT facilitates using local or acute systemic application of smaller doses of the chemotherapeutic, which can reduce side effects, and increases cellular uptake of the chemotherapeutics in the treatment zone (72–74). Cell death via ECT varies based on the chemotherapy's mechanism of action, but reversible electroporation alone does not lead to significant cell death or tumor ablation (75). ECT has been shown to induce apoptosis-like cell death with bleomycin in head and neck carcinoma, necroptosis with bleomycin, cisplatin, and oxaliplatin in pancreatic cancer, and evidence of pyroptosis-like immunogenic cell death when applied with bleomycin in colon cancer (76–78). This is an interesting observation in the variability of cell death mechanisms in different cell types utilizing the same chemotherapeutic. Adjusting chemotherapeutic choice or even dosage for specific malignancies may alter cell death mechanisms and enhance patient outcomes.

Expanding on ECT strategies, the use of calcium represents another option to this treatment strategy. Calcium electroporation enhances necrosis in the treatment zone and its application can forgo or reduce the use of chemotherapeutics and avoid some chemotherapy side effects (79, 80). The induction of necrosis by calcium influx may overcome the chemoresistance mechanisms involving DNA repair and altered survival-linked proteins such as Bcl-2 in some cancers (79, 81–83). Furthermore, there is potential selectivity in calcium electroporation as malignant cells are more likely to die than healthy or benign cells (84, 85). This may be due to the modification many cancers develop to avoid cell death that make them susceptible to calcium imbalances (86, 87).

### Irreversible Electroporation (IRE)

IRE utilizes microsecond pulse electric fields, similar to ECT, but for a higher pulse count. This higher pulse count results in pores and tears forming in the cell membrane that stabilize, leading to loss of cell homeostasis and initiating cell death processes (12, 88). The use of IRE in difficult-to-treat malignancies, such as pancreatic and hepatocellular tumors, is currently becoming more widespread and has led to numerous clinical trials showing inspiringly high success rates (89–95).

**Figure 2:**
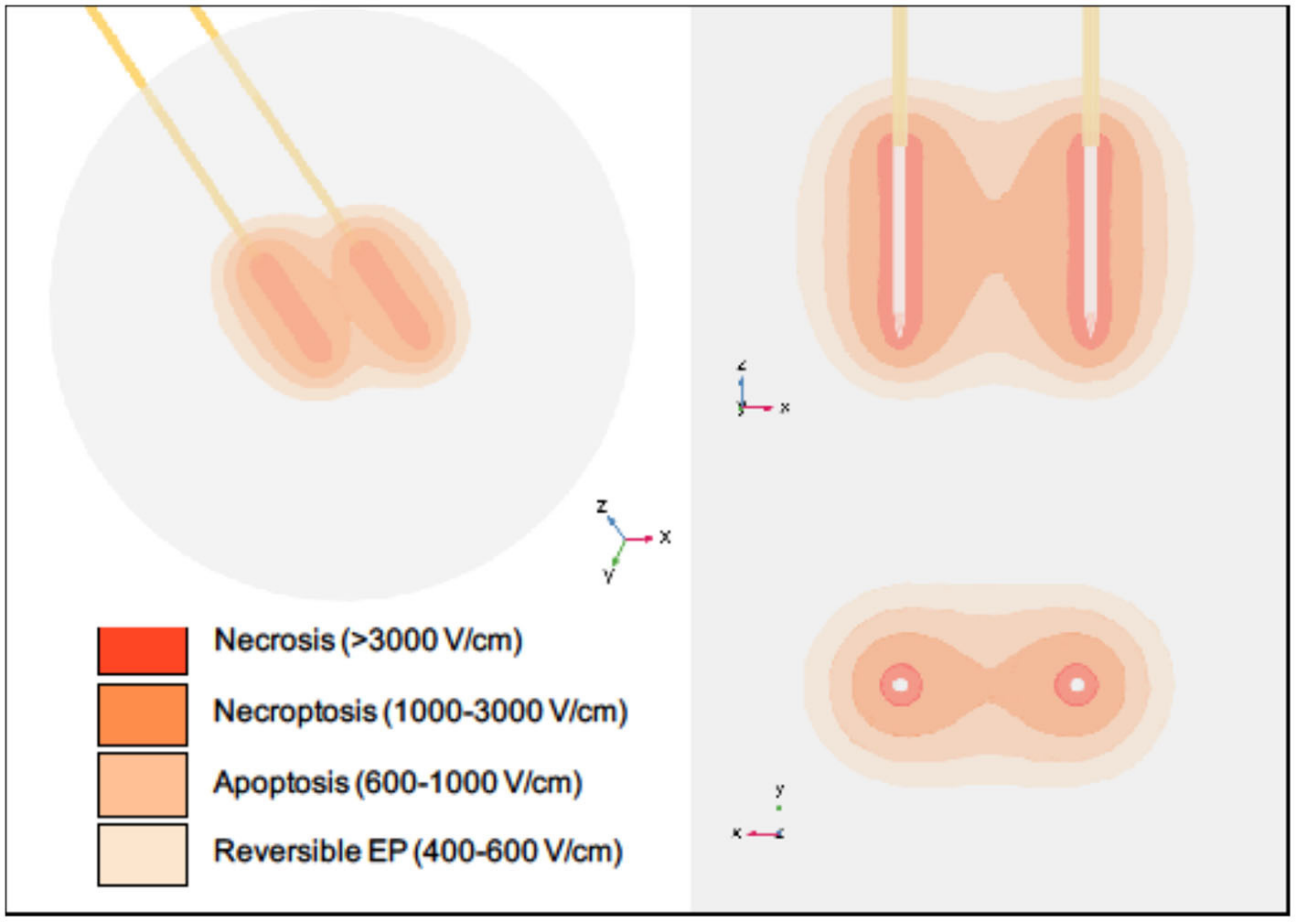
Regions of cell death within a typical IRE treatment zone vary spatially. Necrosis is thought to occur in close proximity to the electrodes during pulse delivery, while necroptosis, apoptosis, and reversible electroporation are thought to occur with increasing distances from the electrodes. It should be noted that this diagram is intended for illustrative purposes only. Specific volumes and thresholds will vary with many parameters, including electrode spacing and exposure, voltage applied, tissue type, number of pulses applied, and time after treatment. The electric field distribution for a single IRE pulse was simulated using COMSOL Multiphysics software (version 5.5, Burlington, MA). Two stainless steel electrodes with insulating holders were inserted into a spherical tissue domain, which was assigned a dynamic electrical conductivity corresponding with normal human pancreatic tissue (97). Electrodes were placed 1.5 cm apart with 1.5 cm exposure, and a voltage of 3,000 V was applied.

IRE-induced cell death was originally considered to be apoptotic (25, 27, 28, 96). However, a majority of these studies did not fully consider alternative cell death types. While apoptosis is certainly occurring in some cells within the treatment zone, our data suggest that treatment could initiate multiple types of cell death mechanisms, though the size and shape of the regions in which each type is experienced may vary between clinical treatments due to differences in pulsing parameters, tissue type, and treatment time [Figure 2; (97, 98)]. This is mediated, in part, by the proximity of the cells to the electrodes, which impacts the voltage each cell experiences during treatment (28, 99–101). While directly near the electrodes may be temperature dependent, cell death becomes temperature independent in other regions based on minimum heating effects seen *in vitro* at comparable electric field magnitudes (7). Multiple studies argued that there may be more than one type of cell death mechanism at play, from necrotic cell death to apoptotic-like non-apoptotic cell death (17, 102–104). These responses could come from differing tissues being predisposed to specific types of cell death depending on the electric field strength applied (105). Likewise, for cells at the margins of the treatment areas, the response may actually be survival signaling to reversible electroporation. In theory, this could be taken advantage of and combined with chemotherapy treatments to increase drug delivery, tumor penetration, and treatment of remnant cancer cells (106–108).

### High-Frequency Irreversible Electroporation (H-FIRE)

Building upon the advantages of IRE, a novel technology termed high-frequency irreversible electroporation (H-FIRE) utilizes 1–10 μs bipolar bursts applied in a pattern with interpulse delay when switching poles. These parameters are highly effective in non-thermally ablating tissues without causing the muscle contractions and heart dysrhythmia associated with the long-duration monophasic pulses of IRE (8, 109–112). Its use in pre-clinical models has progressed without the need for cardiac synchronization as well (112). Cell death following H-FIRE is considered to be highly similar to IRE. However, pre-clinical studies show that H-FIRE may be eliciting immunologic cell death and pyroptosis in addition to apoptosis and necrosis (8, 104). Intriguingly, the use of H-FIRE may allow tuning of cell death with calcium similar to calcium ECT. In *in vitro* hydrogel studies, H-FIRE applied in calcium-rich media showed a significant shift toward necrotic cell death with higher lesion areas and fewer survival signals (113). These data suggest that H-FIRE effects could be modified by injecting calcium or sucrose near the treatment site to allow for controlled applications in difficult-to-treat malignancies or tissue locations.

### Nanosecond Pulsed Electric Fields (nsPEFs)

Nanosecond pulse electric fields (nsPEFs) are characterized by PEFs with short nanosecond pulse durations and high electric fields. Originally, nsPEFs were thought not to permeabilize the cell membrane but rather induce cell death by interfering with molecular patterns inside the cell, disrupting processes in the mitochondria, and other intracellular membrane-bound organelles (114). However, later studies have found that cell membranes and the membranes of internal cellular structures are permeabilized, albeit with smaller tears than those of microsecond PEFs (26, 115–117). nsPEFs have been noted to induce both apoptotic and potentially caspase-independent apoptotic-like cell death similar to necroptosis (116, 117). Recent *in vivo* findings provide greater mechanistic insight into the types of cell death induced by nsPEFs (118–120). Based on the inflammatory and immunomodulatory conditions, these studies reveal that nsPEFs may induce programmed necrosis or necroptotic cell death in breast, melanoma, and pancreatic cancers (118–120).

## Discussion

As electroporation ablation modalities become more mainstream and progress from preclinical studies to clinical applications, characterization of specific cell death mechanisms associated with treatment will become more relevant. The type of cell death has significant consequences for the patient's treatment outcomes, immune system activation, regeneration and repair processes, and co-therapy applications. Seemingly conflicting reports on cell death mechanisms ranging from apoptosis to necrosis and pyroptosis has led to confusion in the field. However, these data suggest that cell death following electroporation may be dependent on electric field and cell type, and that multiple cell death mechanisms could occur within the treatment zone. While studies investigating the effects of related cell lines with varying morphology suggest that cells with larger nuclei are more likely to be affected by H-FIRE, whether these findings are consistent among different electroporation modalities remains to be seen (14, 15). It would be reasonable to hypothesize that different tissues and cell types, both healthy and malignant, have different responses to electroporation-based technologies and may require altering dosing, treatment parameters, or co-therapies to obtain optimal effects. In fact, a recent study on pancreatic cancer shows differences in cell death mechanisms initiated by IRE and HFIRE with similar parameters (104). Further investigation into these cell death mechanisms among electroporation-based technologies may help tailor these treatments for personalized medicine.

## Author Contributions

RB, NB-W, RD, and IA contributed to the writing and editing of the manuscript. All authors contributed to the article and approved the submitted version.

## Conflict of Interest

IA, NB-W, and RD are inventors on pending and issued patents related to the work. The remaining author declares that the research was conducted in the absence of any commercial or financial relationships that could be construed as a potential conflict of interest.
